# Ba_4_RuMn_2_O_10_: A Noncentrosymmetric Polar Crystal Structure with Disordered Trimers

**DOI:** 10.1021/acs.chemmater.4c00586

**Published:** 2024-06-11

**Authors:** Callista M. Skaggs, Peter E. Siegfried, Jun Sang Cho, Yan Xin, V. Ovidiu Garlea, Keith M. Taddei, Hari Bhandari, Mark Croft, Nirmal J. Ghimire, Joon I. Jang, Xiaoyan Tan

**Affiliations:** † Department of Chemistry and Biochemistry, George Mason University, Fairfax, Virginia 22030, United States; ‡ Department of Physics and Astronomy, George Mason University, Fairfax, Virginia 22030, United States; § Quantum Science and Engineering Center, George Mason University, Fairfax, Virginia 22030, United States; ∥ Department of Physics, Sogang University, Seoul 04017, Republic of Korea; ⊥ National High Magnetic Field Laboratory, Florida State University, Tallahassee, Florida 32310, United States; # Neutron Scattering Division, Oak Ridge National Laboratory, Oak Ridge, Tennessee 37831, United States; % X-ray Science Division, Advanced Photon Source, Argonne National Laboratory, Lemont, Illinois 60439, United States; & Department of Physics and Astronomy, Rutgers, The State University of New Jersey, Piscataway, New Jersey 08854, United States; $ Department of Physics and Astronomy and Stavropoulos Center for Complex Quantum Matter, University of Notre Dame, Notre Dame, Indiana 46556, United States

## Abstract

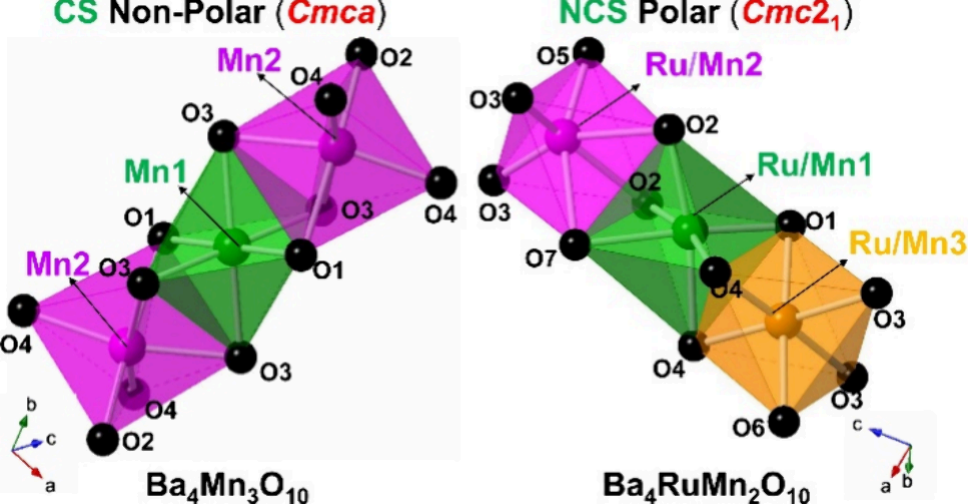

Phase-pure polycrystalline Ba_4_RuMn_2_O_10_ was prepared and determined to adopt the noncentrosymmetric polar crystal structure (space group *Cmc*2_1_) based on results of second harmonic generation, convergent beam electron diffraction, and Rietveld refinements using powder neutron diffraction data. The crystal structure features zigzag chains of corner-shared trimers, which contain three distorted face-sharing octahedra. The three metal sites in the trimers are occupied by disordered Ru/Mn with three different ratios: Ru1:Mn1 = 0.202(8):0.798(8), Ru2:Mn2 = 0.27(1):0.73(1), and Ru3:Mn3 = 0.40(1):0.60(1), successfully lowering the symmetry and inducing the polar crystal structure from the centrosymmetric parent compounds Ba_4_T_3_O_10_ (T = Mn, Ru; space group *Cmca*). The valence state of Ru/Mn is confirmed to be +4 according to X-ray absorption near-edge spectroscopy. Ba_4_RuMn_2_O_10_ is a narrow bandgap (∼0.6 eV) semiconductor exhibiting spin-glass behavior with strong magnetic frustration and antiferromagnetic interactions.

## Introduction

Inorganic materials lacking inversion symmetry are defined as noncentrosymmetric (NCS) materials. A subgroup of NCS crystal structures containing a polar axis is categorized into the NCS polar crystal structure system, which can be simplified as polar materials.^1^ Such materials can be used in laser technology, access memory elements, energy conversion, and spintronics.^1−4^ Currently, it is still very challenging to predict and design novel polar materials based on very limited design strategies.^5−7^ Converting centrosymmetric (CS) compounds into NCS polar materials is an alternative way to achieve polar materials, considering the existence of more CS than NCS materials.^8^ The CS to NCS polar structural transition could happen within a compound as temperature changes, as shown in conventional insulating ferroelectrics. Such ferroelectric-like transitions have also been reported in metallic compounds (e.g., LiOsO_3_), known as interesting “ferroelectric metals”, which have drawn intensive attention in recent years.^9^ We are therefore inspired to achieve NCS polar compounds based CS compounds via small modifications.

**Figure 1:**
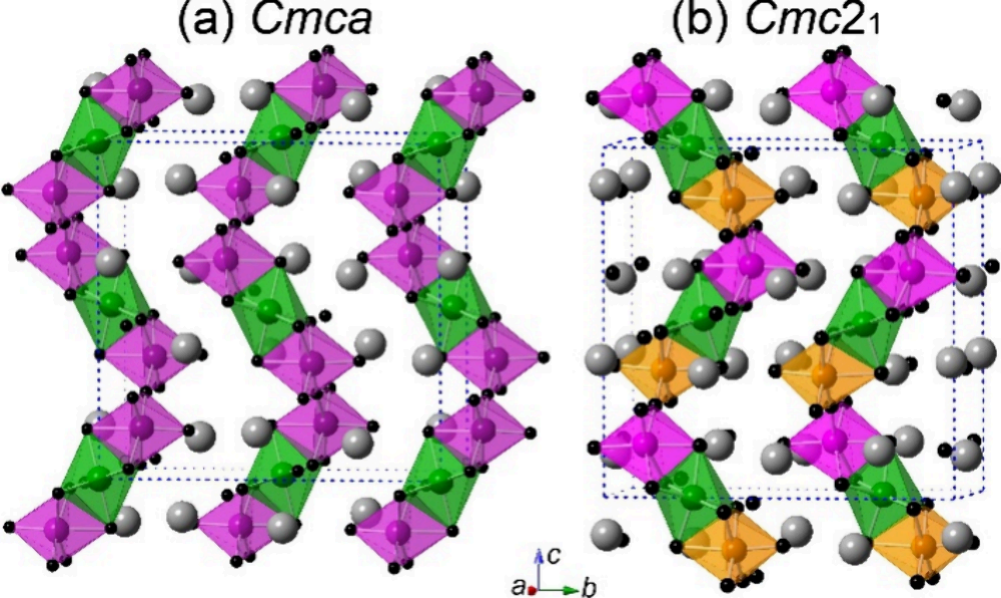
Crystal structure of (a) Ba_4_T_3_O_10_ (T = Mn, Ru) and (b) Ba_4_RuMn_2_O_10_. Color code: Ba = gray, O = black, T1 = green, T2 = purple in (a), Ru/Mn1 = green, Ru/Mn2 = purple, Ru/Mn3 = orange in (b).

Here, we report an interesting example of obtaining NCS polar compounds based on CS parent compounds Ba_4_T_3_O_10_ (T = Mn, Ru). Ba_4_T_3_O_10_ (T = Mn, Ru) fit in the general formula A_*n*+1_T_*n*_O_3*n*+1_ (A = Ca, Sr, Ba, T = transition metals) typically used for representing the Ruddlesden–Popper (R–P) phase. Normal R–P phase crystallizes in the CS space group *I*4/*mmm* featuring corner-sharing octahedra,^10^ while Ba_4_T_3_O_10_ (T = Mn, Ru) adopts a different CS crystal structure (space group *Cmca*) with zigzag chains of corner-shared trimers (T_3_O_12_) containing face-sharing octahedra (Figure 1a).^11−13^ In each trimer, the middle distorted TO_6_ octahedron is symmetrically connected to two identical distorted TO_6_ octahedra, corresponding to the two T sites in the crystal structure. It recently came to our attention that CS Ba_4_T_3_O_10_ (T = Mn, Ru) can be induced to NCS polar compounds by creating three uneven distortions in the trimers, which will promote symmetry breaking to generate a polar crystal structure with three distinct metal sites, lowering the symmetry to *Cmc*2_1_ (a subgroup of the parent *Cmca* space group).^14^ A previous single-crystal X-ray study of Ba_4_Ru_1.1_Mn_1.9_O_10_ indicates that it crystallizes in the polar space group *Cmc*2_1_, with three disordered Ru/Mn sites with different ratios of Ru:Mn on each site (Figure 1b).^15^

To date, the phase-pure polycrystalline sample of Ba_4_Ru_1.1_Mn_1.9_O_10_ has not been reported, and there has been no detailed study to confirm the polar crystal structure via other techniques and to investigate its unknown physical properties further. In this study, we report the synthesis of Ba_4_Ru_1.1_Mn_1.9_O_10_ and the determination of the NCS polar crystal structure with second harmonic generation, convergent beam electron diffraction, and neutron powder diffraction. The oxidation state of transition metals will be revealed with X-ray absorption near-edge spectroscopy. We also report the magnetic and resistivity properties.

## Experimental Section

### Starting Materials and Synthesis

Polycrystalline samples of Ba_4_RuMn_2_O_10_ were synthesized through a two-step solid-state process. BaCO_3_ (99.997% mass fraction, Alfa Aesar), RuO_2_ (99.95% mass fraction, Alfa Aesar), and MnO_2_ (99.997% mass fraction, Alfa Aesar) were used as precursors. The precursors were mixed in a stoichiometric ratio and thoroughly ground in an argon-filled glovebox with a low concentration of O_2_ and H_2_O (<1 ppm). The mixtures were pressed into a 6 mm pellet (<3 tons). The pellet was then placed in an alumina crucible which was heated to 1000 °C in a box furnace over 12 h, retained at the same temperature for 48 h, and cooled to room temperature as the furnace cooled naturally. The obtained sample was then thoroughly ground in air and repressed into a 6 mm pellet (<3 tons), which was heated to 1350 °C over 12 h, held at 1350 °C for 72 h, and cooled to room temperature with furnace cooling. During the sample preparation, no uncommon hazards were noted.

### Powder X-ray and Neutron Diffraction

Powder X-ray diffraction (PXRD) patterns of polycrystalline samples were obtained at room temperature with a scattering angle 2θ ranging from 10° to 70° for 30 min using a benchtop Miniflex-600 powder X-ray diffractometer (Cu Kα, λ = 1.5418 Å). Powder neutron diffraction (PND) data were obtained at the HB-2A (λ = 1.5398 Å) beamline at the High Flux Isotope Reactor at the Oak Ridge National Laboratory.^16,17^ About 3 g of polycrystalline powders were loaded in a vanadium sample holder (inner diameter = 6 mm), and data were collected between 1.7 and 250 K. Rietveld refinements were performed using the FullProf suite of programs.^18^

### Second Harmonic Generation (SHG)

Microcrystalline powders of Ba_4_RuMn_2_O_10_ were sealed in a glass capillary tube and mounted on a homemade sample holder for SHG measurement. Excitation was achieved using an ultrafast Ti:sapphire laser with a pulse width of 100 fs and a repetition rate of 80 MHz, operating at an input wavelength (λ) of 800 nm. The laser beam was focused onto the sample using a lens with a focal length of 75 mm. The signal, with a wavelength (λ_SHG_) of λ/2 (400 nm), was collected under a reflection geometry by using a fiber-optic bundle coupled to a high-resolution spectrometer, which then directed the signal to a charge-coupled device camera. A long data collection time of 10 min was required to obtain the SHG signal well above the signal-to-noise level.

### Chemical Analysis

Elemental analysis of polycrystalline samples of Ba_4_RuMn_2_O_10_ was carried out using a JEOL scanning electron microscope (SEM), JSM-IT500HRLV SEM, and the attached Octane Elect Plus energy dispersive X-ray spectroscopy (EDX) system. The SEM images were collected with an accelerating voltage of 15 kV.

### Transmission Electron Microscopy (TEM)

TEM data were collected on a probe aberration-corrected subangstrom resolution JEOL JEM-ARM200cF microscope at 200 kV. Electron-transparent thin pieces used in the TEM experiments were prepared from crushed polycrystalline powders, which were transferred onto a carbon-coated 200-mesh Cu TEM grid. Selected-area electron diffraction patterns were collected from single thin pieces. Atomic resolution high-angle annular dark-field scanning transmission electron microscopy (HAADF-STEM) images were also obtained using a probe of 0.078 nm with a convergent angle of 21 mrad and an inner collection angle of 74 mrad. Convergent beam electron diffraction (CBED) patterns were obtained by focusing the electron beam onto the sample surface into a 10 nm diameter area in TEM mode.

### X-ray Absorption Near-Edge Spectroscopy (XANES)

The Ru-L_3_ XANES data were collected in total electron yield mode with sequential standards at the National Synchrotron Light Source II (NSLS-II) insertion device beamline 7-ID-2 SST-2 using a Si-111 monochromator, Brookhaven National Laboratory. The Mn-K and Ru-K edge data were collected in both the transmission and fluorescence mode with simultaneous standards at NSLS-II beamline 7BM QAS using a channel cut Si-111 monochromator. Standard spectra were taken on NSLS-I X-19A using a Si-111 double-crystal monochromator.

### Physical Properties

The ACMS II option in a Quantum Design Dynacool physical property measurement system (PPMS) and the MMPS system in the superconducting quantum interference device (SQUID) magnetometer were used to measure the magnetic properties of a polycrystalline sample of Ba_4_Mn_2_RuO_10_. Magnetic susceptibility measurements with zero-field-cooled (ZFC) and field-cooled (FC) modes were recorded between 400 and 1.8 K in a magnetic field of 0.1 and 0.2 T. Field-dependent magnetizations were measured from −9 to 9 T at 2, 10, 50, 100, 200, and 300 K. AC magnetic susceptibility measurements were performed between 1.8 and 25 K with four different frequencies (1, 10, 100, and 1000 Hz) under 0 T. Heat capacity measurements were conducted on a dense pellet of Ba_4_RuMn_2_O_10_ from 1.8 to 50 K under 0 T using PPMS. Resistivity measurement was conducted on a pellet (74% density, diameter = 13 mm) from 300 to 1373 K with the four-probe method using the NETZSCH SBA 458 Nemesis system.

## Results and Discussion

### Synthesis and PXRD

**Figure 2:**
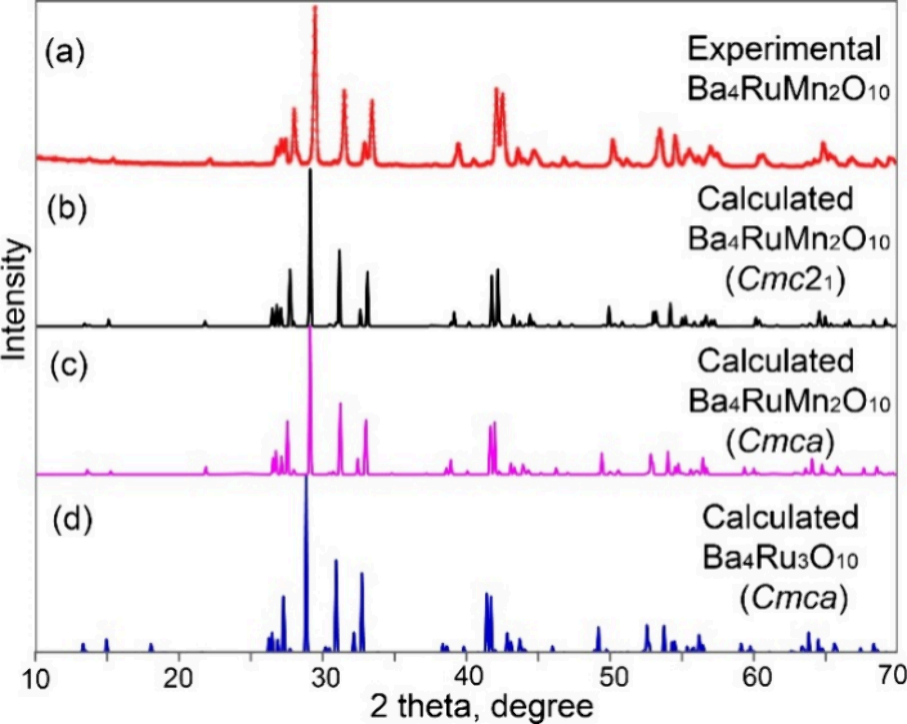
PXRD pattern of Ba_4_RuMn_2_O_10_ (a) compared with calculated patterns of Ba_4_RuMn_2_O_10_ with the space groups *Cmc*2_1_ (b) and *Cmca* (c) and that of Ba_4_Ru_3_O_10_ with the space group *Cmca* (d).

The polycrystalline Ba_4_RuMn_2_O_10_ powders were prepared using a two-step solid-state method with starting materials BaCO_3_, RuO_2_, and MnO_2_ based on a heating profile of Ba_4_Mn_3_O_10_.^19^ The obtained sample was initially checked with a laboratory PXRD technique (Figure 2). The experimental PXRD pattern was compared to the theoretical patterns of Ba_4_RuMn_2_O_10_ (space group *Cmc*2_1_) and parent Ba_4_Ru_3_O_10_ (space group *Cmca*). A calculated pattern of Ba_4_RuMn_2_O_10_ with space group *Cmca* was also simulated based on the structure model of Ba_4_Ru_3_O_10_ with two metal sites via fixing the ratios of Ru:Mn to be 1:2. As shown in Figure S1, three reflections ((020, (021), and (112)) shown at low angles are similar in both theoretical patterns of Ba_4_RuMn_2_O_10_, with a small difference in the intensity of the (020) and (021) reflections. The pattern of Ba_4_Ru_3_O_10_ shows a (111) reflection at low angles, which is not observable in those of Ba_4_RuMn_2_O_10_ but displays a very low intensity of (112) reflection. Our experimental PXRD matches better with the previously reported polar crystal structure with the space group *Cmc*2_1_.

### SHG

**Figure 3:**
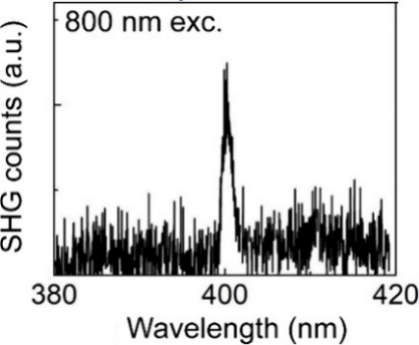
SHG spectrum observed from Ba_4_RuMn_2_O_10_.

To confirm the NCS nature of the crystal structure, an SHG measurement was performed on microcrystalline powders of Ba_4_RuMn_2_O_10_. With an input laser source wavelength (λ) of 800 nm, the SHG signal is observed at a wavelength (λ_SHG_) of λ/2 (400 nm) (Figure 3). The SHG is relatively weak due to the significant absorption of SHG light by the sample itself, considering that the corresponding SHG photon energy (∼3.1 eV) is much higher than the bandgap (∼0.6 eV, as shown below) of Ba_4_RuMn_2_O_10_. The data were carefully collected to ensure that the signal was well above the signal-to-noise level. The SHG signal was confirmed to be intrinsic to the sample, unambiguously demonstrating the NCS nature of Ba_4_RuMn_2_O_10_.

### TEM

**Figure 4:**
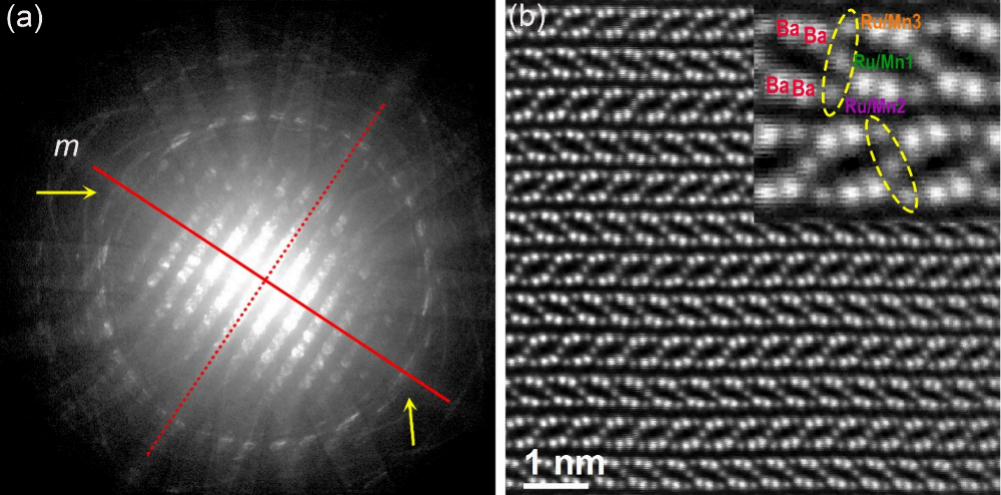
(a) CBED whole pattern along the [010] zone axis. The guiding line for the mirror plane is the solid red line, while the dotted line indicates another possible mirror plane position. The diffraction features that the yellow arrows pointed to are not the same, confirming the nonexistence of a second mirror plane. (b) Atomic resolution HAADF-STEM image of the thin piece along [110] and an enlarged section of HAADF-STEM image (inset) of Ba_4_RuMn_2_O_10_.

To confirm the polar space group (*Cmc*2_1_) of Ba_4_RuMn_2_O_10_, a room-temperature TEM experiment was performed. The CBED pattern is able to distinguish 32 point groups due to dynamic scattering.^20^ The space group *Cmca* has point group *mmm*, while space group *Cmc*2_1_ has point group *mm*2, but both have the same Laue class *mmm*. To distinguish the point group, we tilted the single thin piece to the [010] zone axis and acquired a whole CBED pattern at a low camera length (Figure 4a). For the [010] zone axis, the whole CBED pattern should have 2*mm* symmetry for the point group *mmm* (space group *Cmca*) and one mirror symmetry *m* for the point group *mm*2 (space group *Cmc*2_1_). There is only one mirror plane symmetry *m* for the whole pattern in Figure 4a, confirming that Ba_4_RuMn_2_O_10_ adopts the polar space group *Cmc*2_1_.

The projected atomic structure of the thin piece along the [110] direction is revealed by an atomic resolution high-angle annular dark-field scanning TEM (HAADF-STEM) image (Figure 4b). The intensity of white dots are projected atomic columns in Figure 4b, and they are proportional to the square of atomic number (*Z*^2^) of atoms.^21^ The pairs of intense white dots correspond to two Ba atoms, and the three darker dots between these brighter pairs are projected as three disordered Ru/Mn columns. O atoms (too low *Z*) are not visible in the image. The quantitatively measured intensity for each Ru/Mn column in the HAADF-STEM image is different, suggesting that all three positions are disordered with different ratios of Ru:Mn.

### PND

**Figure 5:**
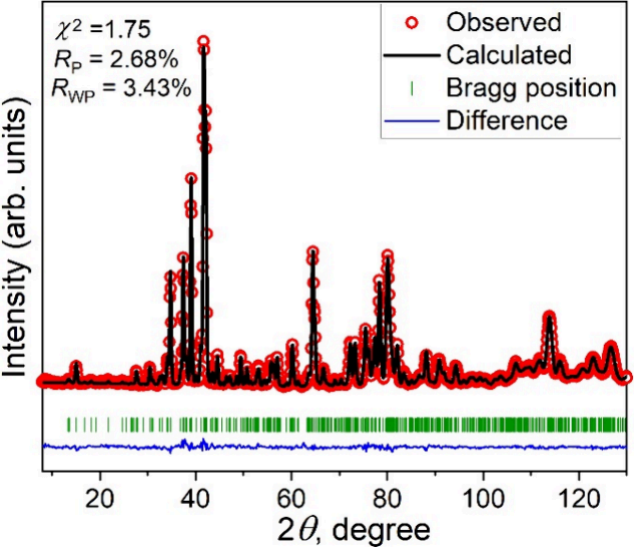
Rietveld refinement of Ba_4_RuMn_2_O_10_ in the space group *Cmc*2_1_ using PND data. The observed data (red), calculated pattern (black), and the difference between those two patterns (blue) are provided.

To further understand the disordered metal sites in the crystal structure, Rietveld refinements were performed using PND data collected at 250 K. The reported crystal structure defined by the space group *Cmc*2_1_ was used as the starting model for Rietveld refinements, which reproduced the experimental PND pattern very well (Figure 5). Selected refined structural parameters are listed in Table 1. The refined unit cell parameters are *a* = 5.7440(1) Å, *b* = 13.1747(3) Å, *c* = 12.8957(2) Å, and *V* = 975.88(3) Å^3^, which are close to the reported values *a* = 5.735(3) Å, *b* = 13.148(4) Å, *c* = 12.855(8) Å, and *V* = 969.3 (8) Å^3^.^4^ The three transition metal sites (T1, T2, and T3) were refined independently with the refined ratios of Ru1:Mn1 = 0.202(8):0.798(8), Ru2:Mn2 = 0.27(1):0.73(1), and Ru3:Mn3 = 0.40(1):0.60(1), which are slightly different from the reported ratios with Ru1:Mn1 = 0.15:0.85, Ru2:Mn2 = 0.325:0.675, and Ru3:Mn3 = 0.6:0.4. Our refined total ratio of Ru:Mn is 0.87:2.13, which is smaller than that of the previously reported Ba_4_Ru_1.1_Mn_1.9_O_10_. This discrepancy can be attributed to small sample differences and data collection of different diffraction techniques, PND vs single-crystal X-ray diffraction. We consider that neutron diffraction offers much better sensitivity to the Ru/Mn atomic ratio due to the large contrast in coherent scattering lengths of Mn (−3.73 fm) and Ru (7.03 fm). To verify the robustness of our reported values, we repeated our Rietveld refinements by varying the number of parameters and also by applying different constraints or restraints to the site occupancies. All refinements produced similar atomic occupancies with Ru:Mn = 0.87:2.1. The chemical analysis using the SEM-EDX technique indicates the ratio of Ba:Ru:Mn:O = 4.1:0.8:1.9:10 (Figure S2), which is close to the ratio of the title compound. For the purpose of simplicity, the nominal composition Ba_4_RuMn_2_O_10_ will be used in the following discussion.

**Table 1:** Selected Structure Parameters of Rietveld Refinement Details of Ba_4_RuMn_*2*_O_10_ Using PND Data

sample	Ba_4_RuMn_2_O_10_
empirical formula	Ba_4_Ru0.87(2)Mn2.13(2)O_10_
temperature, K	250
mol wt, g/mol	914.25
density (calculated), g/cm^3^	6.225
neutron wavelength (λ), Å	1.5398
space group, #	*Cmc*2_1_, #36
*Z*	4
lattice parameters	*a* = 5.7440(1) Å, *b* = 13.1747(3) Å, *c* = 12.8957(2) Å, *V* = 975.88(3) Å^3^
Rietveld criteria of fit	*R*_p_ = 2.68%, *R*_wp_ = 3.43%, *R*_Bragg_ = 3.44%, χ^2^ = 1.75

site	Wyckoff symbol	*x*, *y*, *z*	occ
Ba1	4*a*	0, 0.006(1), 0.907(2)	1
Ba2	4*a*	0, 0.717(2), 0.869(2)	1
Ba3	4*a*	0, 0.214(1), 0.649(2)	1
Ba4	4*a*	0, 0.511(1), 0.624(2)	1
Ru1/Mn1	4*a*	0, 0.240(3), 0	0.202(8)/0.798(8)
Ru2/Mn2	4*a*	0, 0.882(5), 0.678(5)	0.27(1)/0.73(1)
Ru3/Mn3	4*a*	0, 0.362(7), 0.867(3)	0.40(1)/0.60(1)
O1	4*a*	0, 0.215(1), 0.864(2)	1
O2	8*b*	0.224(1), 0.1470(8), 0.047(2)	1
O3	8*b*	0.253(3), 0.3692(3), 0.756(2)	1
O4	8*b*	0.221(2), 0.3564(8), 0.975(2)	1
O5	4*a*	0, 0.0148(1), 0.649(2)	1
O6	4*a*	0.5, 0.015(1), 0.860(2)	1
O7	4*a*	0.5, 0.220(1), 0.655(2)	1

**Figure 6:**
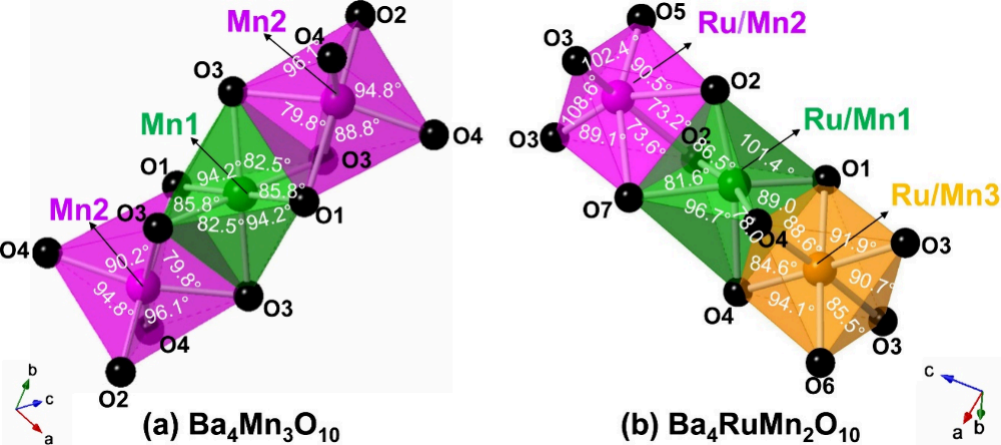
A zoomed-in trimer in the crystal structure of (a) Ba_4_Mn_3_O_10_ (space group *Cmca*) and (b) Ba_4_RuMn_2_O_10_ (space group *Cmc*2_1_). Color code: Ru/Mn1 = green, Ru/Mn2 = purple, Ru/Mn3 = orange, O = black.

The refined Mn/Ru–O bond distances (*d*) vary from 1.79(3) to 2.15(7) Å (Table 2), which are close to those (1.83–2.14 Å) in Ba_4_Ru_1.1_Mn_1.9_O_10_,^15^ the Ru–O bond distances (1.92–2.12 Å) in Ba_4_Ru_3_O_10_,^22^ and the Mn–O bond distances (1.81–2.09 Å) in Ba_4_Mn_3_O_10_.^12^Figure 6 shows a comparison of a zoomed-in trimer of Ba_4_Mn_3_O_10_ and Ba_4_RuMn_2_O_10_. In the trimer of Ba_4_Mn_3_O_10_ (Figure 6a), the centering Mn1O_6_ octahedron is more symmetric than the Mn_2_O_6_ octahedra: Mn1 atom coordinates with four O3 (*d* = 1.90 Å) and two O1 (*d* = 1.91 Å), and the angles of O–Mn1–O are 82.5°, 85.8°, and 94.2°, while the Mn2 atom coordinates with two O3 (*d* = 1.96 Å), two O4 (*d* = 1.88 Å), and one O2 (*d* = 1.81 Å) and O1 (*d* = 2.09 Å), and the angles of O–Mn2–O vary from 79.8° to 96.1°.^12^ The trimer of Ba_4_RuMn_2_O_10_ is more distorted than that of Ba_4_Mn_3_O_10_ due to a larger difference in bond distances within each octahedron (Table 2), with the ranges of 1.79–2.07, 1.79–2.15, and 1.89–2.04 Å in the Ru/Mn1O_6_, Ru/Mn_2_O_6_, and Ru/Mn_3_O_6_ octahedron, respectively. The angles of the octahedrons of the O–Ru/Mn–O vary in the ranges of 78.0°–101.4°, 73.2°–108.6°, and 85.5°–94.1° in the Ru/Mn1O_6_, Ru/Mn_2_O_6_, and Ru/Mn_3_O_6_ octahedron, respectively (Figure 6b). The Ru/Mn3O_6_ octahedron is the least distorted and the Ru/Mn2O_6_ octahedron is the most distorted in the trimer of Ba_4_RuMn_2_O_10_.

**Table 2:** Selected Bond Distances in Ba_4_RuMn_2_O_10_

	Ru/Mn1–O distances (Å)	Ru/Mn2–O distances (Å)			Ru/Mn3–O distances (Å)
Ru/Mn1–O1	1.79(3)	Ru/Mn2–O5	1.79(7)	Ru/Mn3–O4	1.89(4) × 2
Ru/Mn1–O2	1.88(3) × 2	Ru/Mn2–O3	1.75(5) × 2	Ru/Mn3–O6	2.02(9)
Ru/Mn1–O4	2.02(3) × 2	Ru/Mn2–O2	2.16(6) × 2	Ru/Mn3–O1	1.94(9)
Ru/Mn1–O7	2.07(3)	Ru/Mn2–O7	2.15(7)	Ru/Mn3–O3	2.04(4) × 2

The three partially disordered metal sites in Ba_4_RuMn_2_O_10_ are different from the totally disordered sites, where two elements are randomly occupied with a ratio of 50:50. Many double perovskite A_2_B′B″O_6_ (A, B = elements as cation ions) form totally disordered sites when the radius difference (Δ*r*) of B′ and B″ ions is within 0.04 Å.^23^ The formation of such partially disordered metal sites in Ba_4_RuMn_2_O_10_ may be related to the large Δ*r* (0.09 Å) between 3*d*-Mn^4+^ (0.53 Å) and 4*d*-Ru^4+^ ions (0.62 Å).^24^ The designing strategy, creating unevenly disordered sites in trimers via mixing two different transition metal ions with a relatively large Δ*r*, is different from the typical method of stabilizing polar materials with ordered cation ions, e.g., A_2_B′B″O_6_,^25−29^ Ba_4_RuTi_2_O_9_,^30,31^ Ba_3_CuSb_2_O_9_,^32^ etc. But it is worth noting that polar materials with partially disordered sites have been reported in some hexagonal perovskite-related compounds containing dimers, such as Ba_3_Fe_1.56_Ir_1.44_O_9_ (*P*3_1_*mc*)^33^ and Ba_3_TiIr_2_O_9_ (*P*6_3_*mc*).^34,35^ Ba_4_RuMn_2_O_10_ is an interesting example of converting CS materials with trimers to NCS polar materials.

### XANES

Based on the experimental bond distances in Table 2, the bond valence sum (BVS) can be estimated for each site^36^ by assuming the T1 = Mn, T2 = Mn, T3 = Ru, and the calculated BVS value for each T site is 3.8, 4.0, and 4.2, respectively, which indicates the oxidation state of +4 for Ru/Mn ions. To further study the valence states of Ru and Mn, XANES measurements were performed to collect the Mn-K, Ru-L_3_, and Ru-K edges.

**Figure 7:**
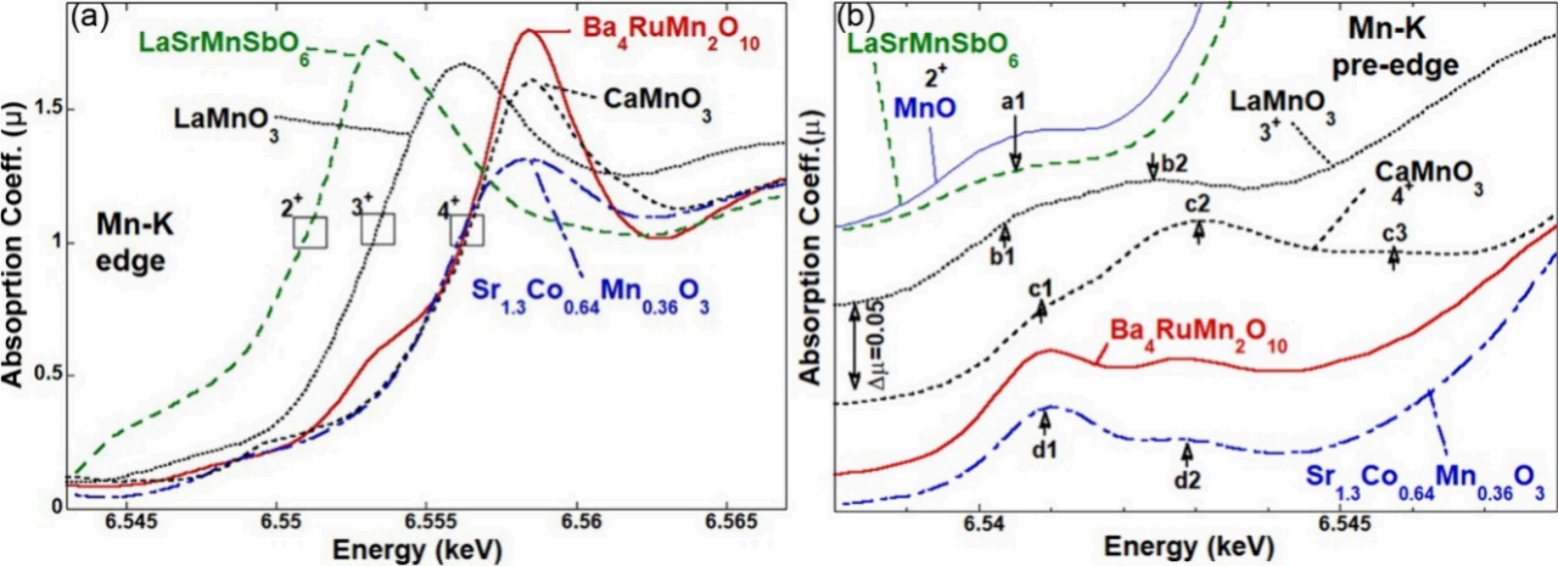
(a) Mn–K edge and (b) pre-edge spectra of Ba_4_RuMn_2_O_10_ and a series of standard Mn-containing compounds: LaSrMn^2+^SbO_6_, LaMn^3+^O_3_, and CaMn^4+^O_3_.

The main edge features at 3*d* transition metal K edges are dominated by 1*s* to 4*p* transition peak features, riding on a step-continuum feature, with a chemical shift to higher energy with increasing valence. In comparison with perovskite-based (corner sharing) LaSrMn^2+^SbO_6_, LaMn^3+^O_3_, and CaMn^4+^O_3_, and face-sharing Sr_1.3_Co_0.64_Mn^4+^O_3_ standards,^37−39^ the chemical shift of the Mn–K edge of Ba_4_RuMn_2_O_10_ compounds is consistent with a Mn^4+^ valence assignment (Figure 7a). Moreover, the lower energy shoulder in the title compound spectrum (near 6.553 keV) is similar to that of the face-sharing standard spectrum of Sr_1.3_Co_0.64_Mn^4+^O_3_.^39^

The pre-edge features provide additional insight into the valence state. As shown in Figure 7b, the a1, b1–b2, and c1–c2–c3 features are characteristic of the non-face-sharing Mn^2+^, Mn^3+^, and Mn^4+^ in LaSrMn^2+^SbO_6_, LaMn^3+^O_3_, and CaMn^4+^O_3_, respectively. The d1–d2 feature pattern is characteristic of face-sharing Mn^4+^ in the Sr_1.3_Co_0.64_Mn^4+^O_3_. The pre-edge spectrum of Ba_4_RuMn_2_O_10_ is consistent with that of Sr_1.3_Co_0.64_Mn^4+^O_3_ (Figure 7b), which further confirms the face-sharing Mn^4+^ character of this compound.

**Figure 8:**
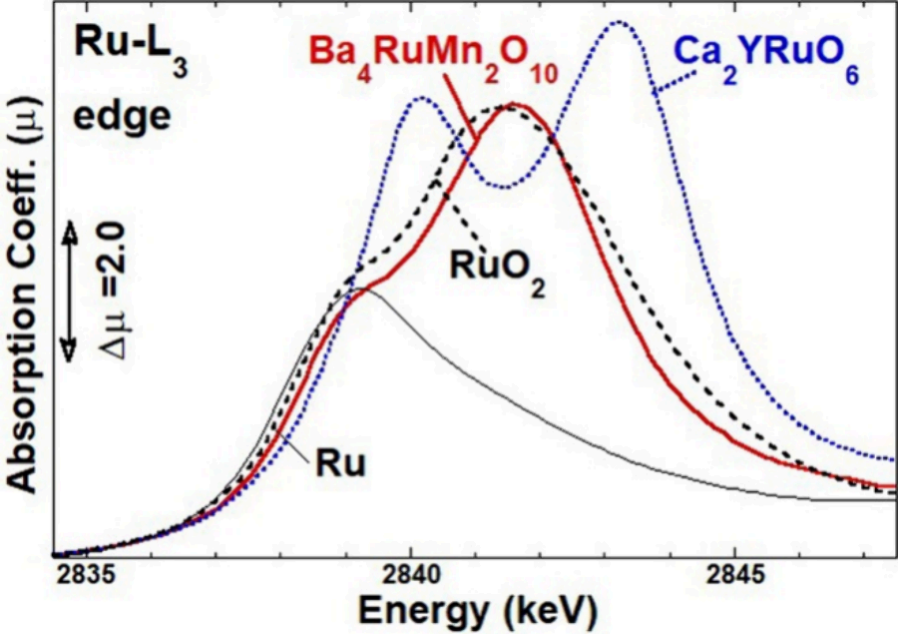
(a) Ru-L_3_ edges of Ba_4_RuMn_2_O_10_ and a set of standard materials: Ru, RuO_2_, and Ca_2_YRuO_6_.

The Ru-L_3_ edge of Ba_4_RuMn_2_O_10_ is compared with those of the elemental Ru^0^, Ru^4+^O_2_, and Ca_2_YRu^5+^O_6_ standards (Figure 8).^40,41^ The Ru-L_3_ edge is dominated by an intense peak feature due to transitions into empty 4*d* states with the peak structures directly reflecting the 4*d*-orbital crystal field splittings. With increasing valence (decreasing 4*d* count) in the elemental Ru^0^-4*d*^7^, Ru^4+^-4*d*^4^, and Ru^5+^-4*d*^3^, one can clearly note that the centrum of these 4*d*-features chemically shifts to higher energy, and its intensity increases (due to the presence of additional 4*d* hole states).^40,41^ The centrum chemical shift and the spectral intensity (and distribution) of the Ba_4_RuMn_2_O_10_ Ru-L_3_ spectrum evidence its Ru^4+^-4*d*^4^ configuration assignment. This assignment is corroborated by the chemical shift of the Ru–K edge of Ba_4_RuMn_2_O_10_ compared to those of Ru^0^, Ru^4+^O_2_, Ca_2_YRu^5+^O_6_, and Sr_2_YRu^5+^O_6_ in Figure S3.

### Physical Properties

**Figure 9:**
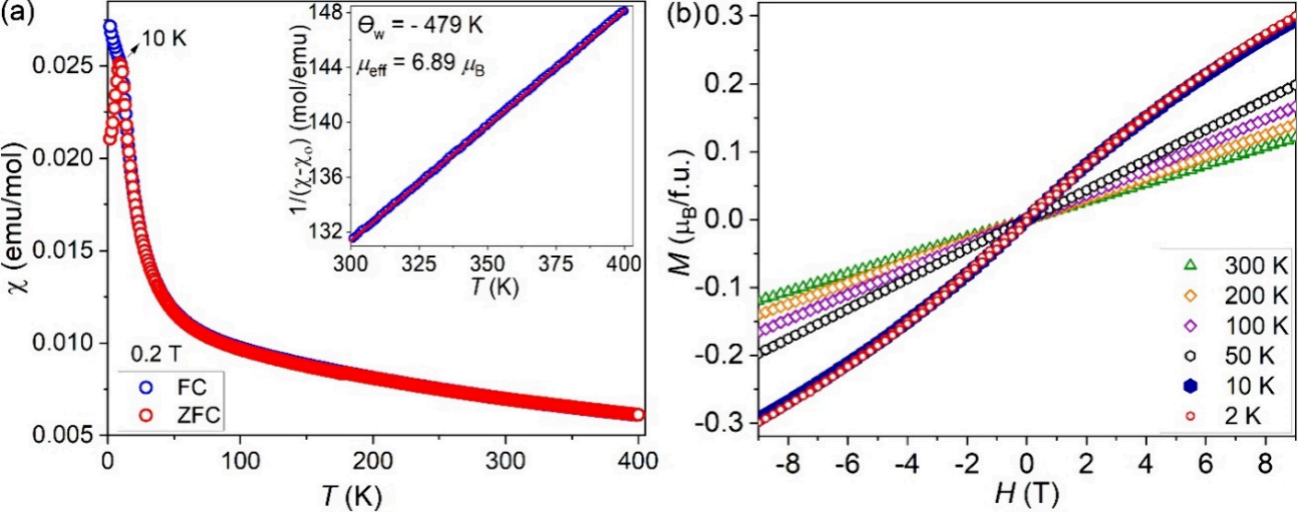
(a) ZFC-FC magnetic susceptibility with a magnetic field of 0.2 T with inverse FC magnetic susceptibility fit of Ba_4_RuMn_2_O_10_ with the 1/(χ – χ_0_) = (*T* – Θ_w_)/*C* equation (inset) and (b) isothermal-field-dependent magnetization of Ba_4_RuMn_2_O_10_ between 2 and 300 K (inset).

The zero-field-cooled and field-cooled (ZFC-FC) magnetic susceptibility data present a cusp near 10 K in the ZFC process, but it keeps increasing in the FC process. The observed divergence between the ZFC and FC data indicates the possible spin-glass behavior (Figure 9). For spin-glass materials, the FC magnetic susceptibility typically saturates due to spin freezing below the defined temperature *T*_f_, the cusp that is more obvious in the ZFC process. In this compound, the FC susceptibility shows behavior similar to that of ZFC when the applied magnetic field is 0.1 T (Figure S4), but it increases obviously below the *T*_f_ (10 K) when the magnetic field is increased to 0.2 T, which supports the metastable frozen states in the spin-glass materials. The isothermal field-dependent magnetization data measured at 10 K overlap with those at 2 K, and it slightly deviates from the linear behavior (Figure 9b). The magnetizations measured between 50 and 300 K are linear, as expected. Fitting the inverse FC magnetic susceptibility between 300 and 400 K with the Curie–Weiss law, χ = χ_0_ + *C*/(*T* – Θ_w_), yields the constant diamagnetic term χ_0_ = −0.0006(2) emu/mol and a negative Weiss constant (Θ_w_) of −479 K (Figure 9a), which indicates the antiferromagnetic (AFM) interactions. The obtained effective moment (μ_eff_ = 6.89 μ_B_/f.u.) given by , where *C* is the Curie constant from the Curie–Weiss fitting, is smaller than the expected theoretical value (7.27 μ_B_/f.u.) based on μ_eff_^2^*=* 0.87 × μ_eff_(Ru^4+^)^2^ + 2.13 × μ_eff_(Mn^4+^)^2^, with μ_eff_(Ru^4+^) = 4.9 μ_B_ and μ_eff_(Mn^4+^) = 3.87 μ_B_. The calculation of the frustration factor (*f*) with the formula *f* = |Θ_w_|/*T*_f_ yields *f* = 48, which indicates the existence of strong magnetic frustration that could be related to the disordered Ru/Mn site in the trimers.

The low-temperature neutron diffraction data were collected, but no additional scattering associated with the long-range magnetic ordering was detected (Figure S5), suggesting strongly fluctuating moments of a glassy magnetic ground state. The magnetic behavior of Ba_4_RuMn_2_O_10_ is different from the long-range antiferromagnetic (AFM) ordering observed in the parent compounds, Ba_4_Ru_3_O_10_ and Ba_4_Mn_3_O_10_, which shows a Néel temperature (*T*_N_) at ∼105 and ∼40–80 K, respectively.^12,19,42,43^ In the magnetic structure of Ba_4_Ru_3_O_10_, only Ru2 sites (Ru atoms at the end of the trimer) order magnetically with ∼1 μ_B_ in an antiparallel arrangement (Ru1 site remains paramagnetic), while all Mn atoms are ordered with a larger magnitude of moment (2.23 and −1.98 μ_B_) in the Ba_4_Mn_3_O_10_ compound.^12,43^ If we assume there are AFM states in Ba_4_RuMn_2_O_10_ adopting the magnetic structure of parent compounds in such a polar crystal structure (Figure S6), fitting the 1.7 K data with adapted models yields reduced ordered moments per mixed Mn/Ru site of 0.4(1) μ_B_ (Figure S7). This moment represents our detection limit defined by our experimental error bars.

**Figure 10:**
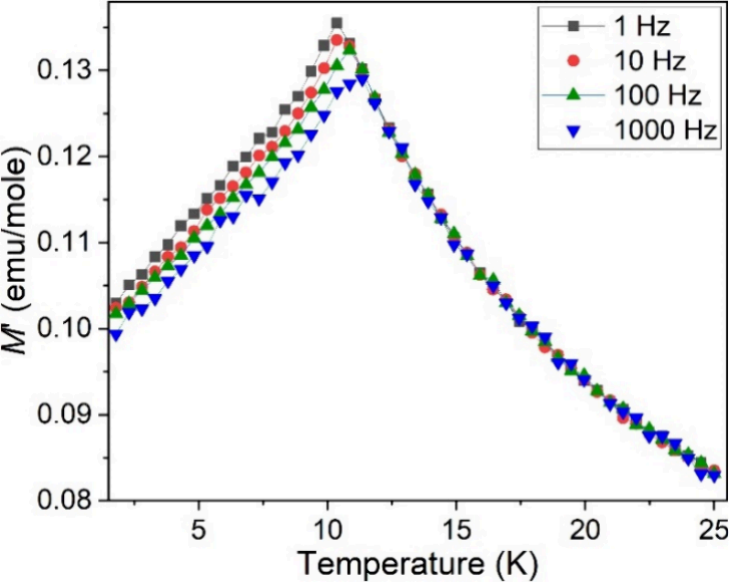
AC magnetic susceptibility data of Ba_4_RuMn_2_O_10_.

To confirm the spin-glass behavior, AC magnetic susceptibility measurements were carried out at four different frequencies (1, 10, 100, and 1000 Hz), showing typical frequency-dependent maxima near the spin-freezing transition temperature (Figure 10). The empirical Mydosh parameter (φ) can be calculated based on φ = (*T*_max_(ν_1_) – *T*_max_(ν_2_))/(*T*_max_(ν_1_) × (log ν_1_ – log ν_2_)), where the *T*_max_(ν) is the temperature corresponding to the maxima in the AC magnetic susceptibility at the frequency of ν. The calculated φ is 0.032(1), which is within the typical range (0.004–0.08) of spin glasses.^44^ The spin glass behavior is probably due to the disordered metal sites and strong magnetic frustration in this system.

**Figure 11:**
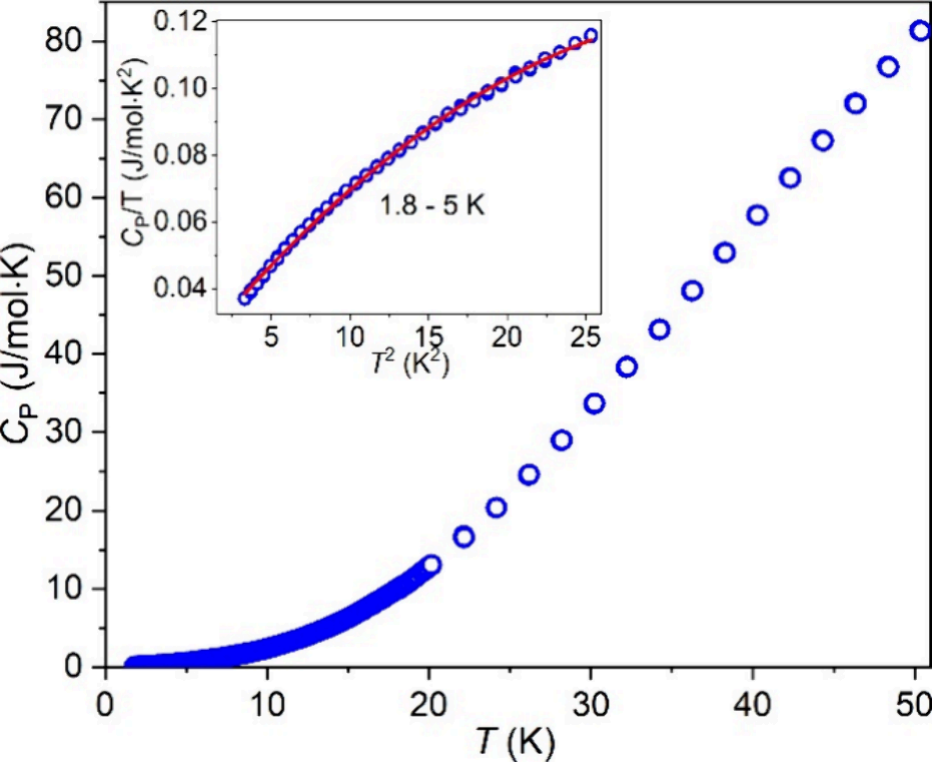
Heat capacity *C*_P_ measured from 1.8 to 50 K (the low-temperature range) fitting with the modified Debye model *C*_P_/*T* = γ + β*T*^2^+ α*T*^4^ (inset).

Heat capacity (*C*_P_) measurements were conducted on a dense pellet of Ba_4_RuMn_2_O_10_ from 1.8 to 50 K under 0 T (Figure 11). The absence of a typical λ-like anomaly supports the lack of long-range magnetic ordering. The heat capacity *C*_P_ has both electron (*C*_e_ = γ*T*) and lattice contribution (*C*_l_ = β*T*^3^+ α*T*^5^). For this compound (not metallic as shown below), the *C*_l_ is much larger than *C*_e_, and *C*_P_ requires three terms in the modified Debye model (*C*_P_/*T* = γ + β*T*^2^+ α*T*^4^) to fit the low-temperature range (1.8–5 K), which yields the Sommerfeld coefficient γ (21 mJ/(mol·K^2^)) and coefficient β (5.67 × 10^–3^ J/(mol·K^4^)). The obtained γ (21 mJ/(mol·K^2^)) is comparable with the value obtained in the related compound Ba_4_Ir_3_O_10_, which also lacks the long-range magnetic order but shows strong AFM exchange interactions.^45^ The coefficient β is associated with the Debye temperature (Θ_D_) as Θ_D_^3^ = 12π^4^*Rn*/(5β), where *R* = 8.314 J/(mol·K), *n* = 17 (the number of atoms per formula unit), and the Θ_D_ is determined to be 180 K, which is smaller than that (400 K) of Ba_4_Mn_3_O_10_.^46^

**Figure 12:**
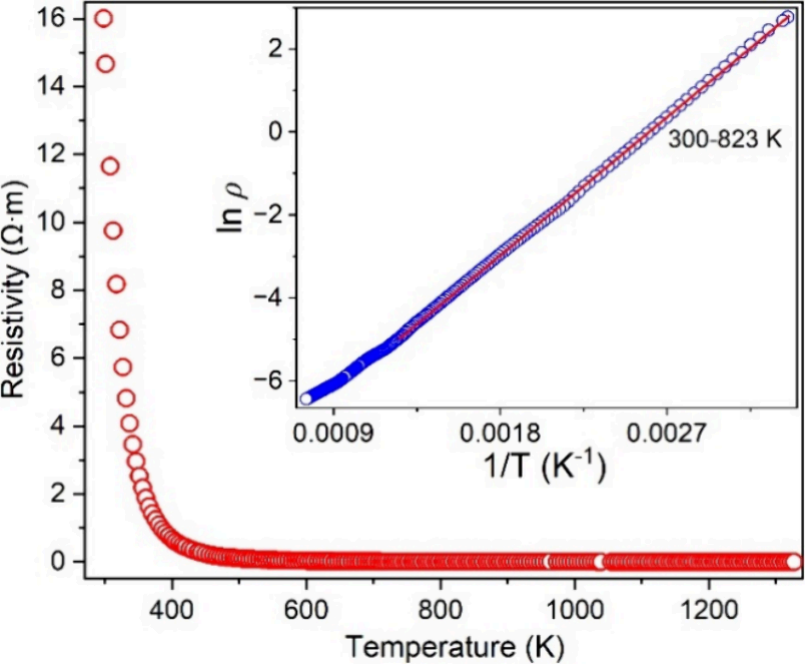
Temperature-dependent resistivity of Ba_4_RuMn_2_O_10_ with a plot of ln ρ vs 1/*T* with the linear fitting of the data (300–823 K) using the Arrhenius equation ln ρ = ln ρ_0_ + *E*_a_/*k*_B_*T*.

Resistivity measured as a function of temperature (300–1373 K) indicates that it is a semiconductor. The bandgap (*E*_g_) can be estimated via the formula of *E*_g_ ∼ 2*E*_a_ (thermal activation energy) and the fitting of the data with the Arrhenius equation ln ρ = ln ρ_0_ + *E*_a_/*k*_B_*T*, where ρ_0_ is the pre-exponential factor and *k*_B_ is the Boltzmann constant (Figure 12). Based on the obtained value of *E*_a_, the *E*_g_ is estimated to be ∼0.6 eV, which is close to those theoretical values of Ba_4_Ru_3_O_10_.^47,48^

## Conclusion

In summary, phase-pure polycrystalline Ba_4_RuMn_2_O_10_ can be successfully prepared via the high-temperature solid-state method. The NCS polar space group was confirmed by SHG, CBED, and HAADF-STEM. Rietveld refinements using PND data proved the three partially disordered Ru/Mn sites with different ratios of Ru:Mn. XANES confirms the +4 valence state of Ru/Mn. Ba_4_RuMn_2_O_10_ exhibits semiconducting behavior with a narrow bandgap and spin-glass state. The design strategy of converting the CS Ba_4_T_3_O_10_ (T = Mn, Ru) into NCS polar Ba_4_RuMn_2_O_10_ via creating three unevenly distorted octahedra in the trimers with partially disordered Ru/Mn metal sites is successful. This method may be applied to prepare Ba_4_Ru_3–*x*_Mn_*x*_O_10_ (0 < *x* < 3) and other novel compounds based on Ba_4_T_3_O_10_ (T = Mn, Ru, Ir) parent compounds and similar CS crystal structures featuring trimers or pentamers.

## Supporting Information Available

XRD patterns at low angles; EDX elemental maps; Ru-K edges of Ba_4_RuMn_2_O_10_; ZFC-FC data at 0.1 T; PND patterns of Ba_4_RuMn_2_O_10_ at 1.7 and 250 K; magnetic structures of the parent compounds redefined for the polar symmetry of Ba_4_RuMn_2_O_10_; the refined neutron data of Ba_4_RuMn_2_O_10_ measured at 1.7 K (PDF)
