# Supplementary material for: Ba4RuMn2O10: A Noncentrosymmetric Polar Crystal Structure with Disordered Trimers
Source: Chem Mater. 2024 Jun 11;36(12):6053–61. doi: 10.1021/acs.chemmater.4c00586 (PMC11210430; doi:10.1021/acs.chemmater.4c00586)
Supplement: Supplementary file 1 — cm4c00586_si_001.pdf [file cm4c00586_si_001.pdf]

$Ba_4RuMn_2O_{10}$: A Noncentrosymmetric Polar Crystal Structure with Disordered Trimers

Callista M. Skaggs,[†] Peter E. Siegfried,[‡,§] Jun Sang Cho,[‖] Yan Xin,[⊥] V. Ovidiu Garlea,[#] Keith M. Taddei,[#,%] Hari Bhandari, [‡,$] Mark Croft,[&] Nirmal J. Ghimire,[$] Joon I. Jang,[‖] Xiaoyan Tan*[1,§]

[†]*Department of Chemistry and Biochemistry, George Mason University, Fairfax, Virginia 22030, United States*
[‡]*Department of Physics and Astronomy, George Mason University, Fairfax, Virginia 22030, United States*
[§]*Quantum Science and Engineering Center, George Mason University, Fairfax, Virginia 22030, United States*
[‖]*Department of Physics, Sogang University, Seoul 04017, Republic of Korea*
[⊥]*National High Magnetic Field Laboratory, Florida State University, Tallahassee, Florida 32310, United States*
[#]*Neutron Scattering Division, Oak Ridge National Laboratory, Oak Ridge, Tennessee 37831, United States*
[%]*X-ray Science Division, Advanced Photon Source, Argonne National Laboratory, Lemont, Illinois 60439, United States*
[&]*Department of Physics and Astronomy, Rutgers, The State University of New Jersey, Piscataway, New Jersey 08854, United States*
[$]*Department of Physics and Astronomy and Stavropoulos Center for Complex Quantum Matter, University of Notre Dame, Notre Dame, Indiana 46556, United States*

Corresponding Authors* Email: xtan6@gmu.edu

Page

Figure S1. The PXRD pattern (10°-30°) of $Ba_4RuMn_2O_{10}$ (a) compared with calculated patterns of $Ba_4RuMn_2O_{10}$ with the space groups *Cmc*2₁ (b) and *Cmca* (c), and that of $Ba_4Ru_3O_{10}$ with space group *Cmca* (d) S2
Figure S2. Energy-dispersive X-ray spectroscopy (EDX) elemental maps of $Ba_4RuMn_2O_{10}$ particles. S2
Figure S3. The Ru-K edges of $Ba_4RuMn_2O_{10}$ and a set of standard materials: elemental-Ru^0, and octahedrally coordinated $Ru^{4+}O_2$ (edge-sharing chains) and perovskite-based $Ca_2YRu^{5+}O_6$ and $Sr_2YRu^{5+}O_6$ (corner-sharing) S2
Figure S4. ZFC-FC magnetic susceptibility with a magnetic field of 0.1 T. S3
Figure S5. Powder neutron diffraction patterns of $Ba_4RuMn_2O_{10}$ at 1.7 and 250 K S3
Figure S6. Magnetic structures reported for the parent compounds $Ba_4Ru_3O_{10}$ (a) and $Ba_4Mn_3O_{10}$ (b) redefined for the polar symmetry of the $Ba_4RuMn_2O_{10}$ S4
Figure S7. (a) The refined neutron data of $Ba_4RuMn_2O_1$ measured at 1.7 K, assuming only a nuclear contribution (green line in a) and low angle ranges of simulated magnetic Bragg intensities for the two parent compounds $Ba_4Ru_3O_{10}$ (red line in b) and $Ba_4Mn_3O_{10}$ (blue line in c) with the adopted polar crystal structure of $Ba_4RuMn_2O_{10}$. S4

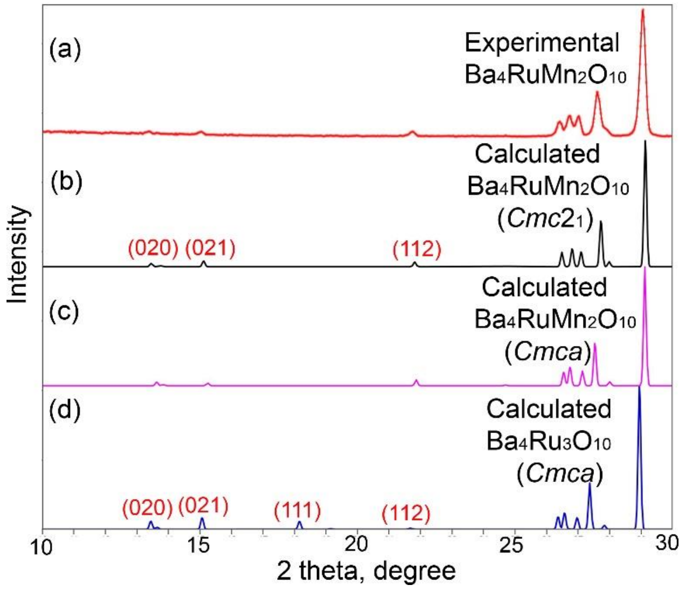

Figure S1. PXRD pattern (10°-30°) of $Ba_4RuMn_2O_{10}$ (a) compared with calculated patterns of $Ba_4RuMn_2O_{10}$ with the space groups *Cmc2*$_1$ (b) and *Cmca* (c), and that of $Ba_4Ru_3O_{10}$ with the space group *Cmca* (d).

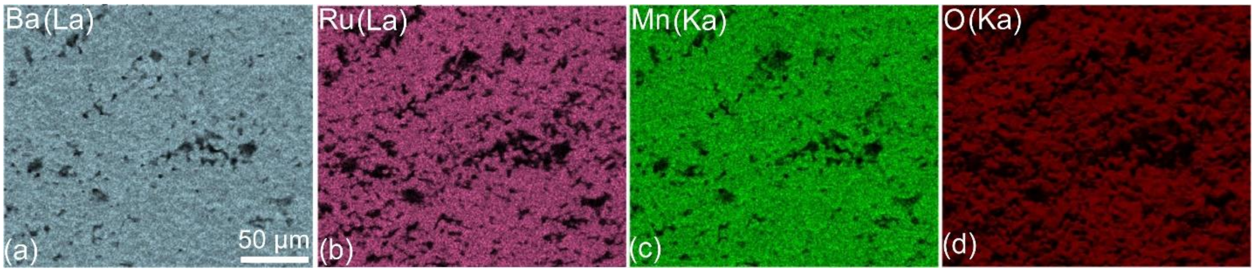

Figure S2. Energy-dispersive X-ray spectroscopy (EDX) elemental maps of $Ba_4RuMn_2O_{10}$ particles.

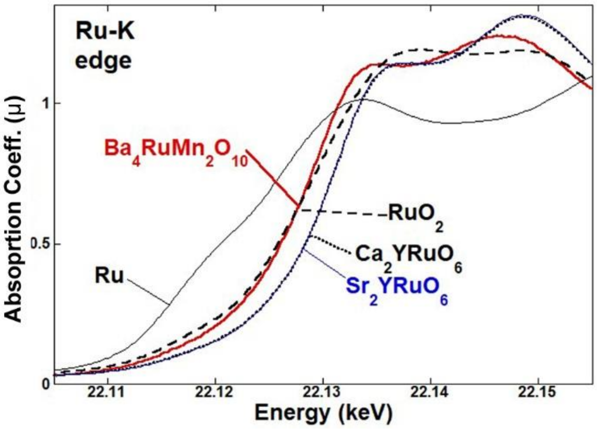

Figure S3. The Ru-K edges of $Ba_4RuMn_2O_{10}$ and a set of standard materials: elemental-Ru^0, and octahedrally coordinated $Ru^{4+}O_2$ (edge-sharing chains) and perovskite-based $Ca_2YRu^{5+}O_6$ and $Sr_2YRu^{5+}O_6$ (corner-sharing). Note that the chemical shift of the $Ba_4RuMn_2O_{10}$ spectrum confirms its Ru^{4+} configuration.

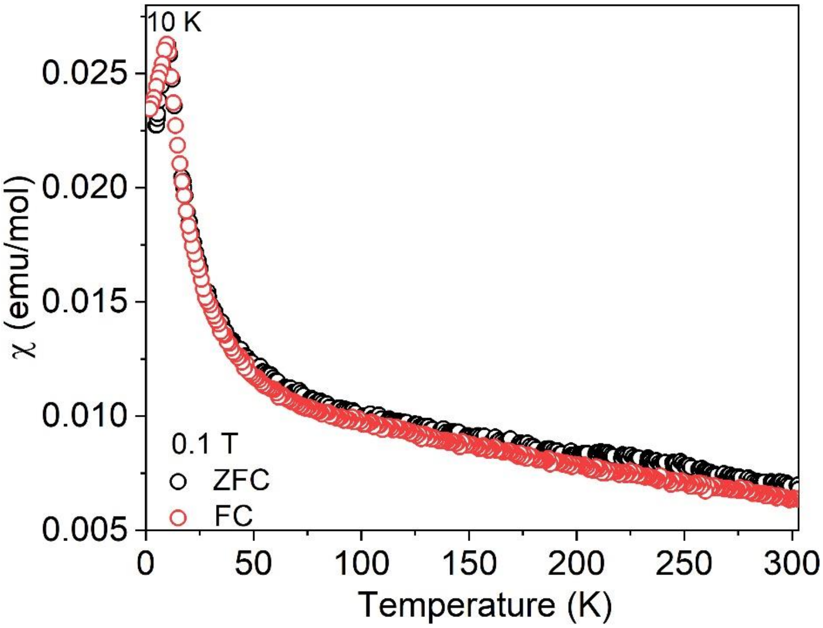

Figure S4: ZFC-FC magnetic susceptibility with a magnetic field of 0.1 T.

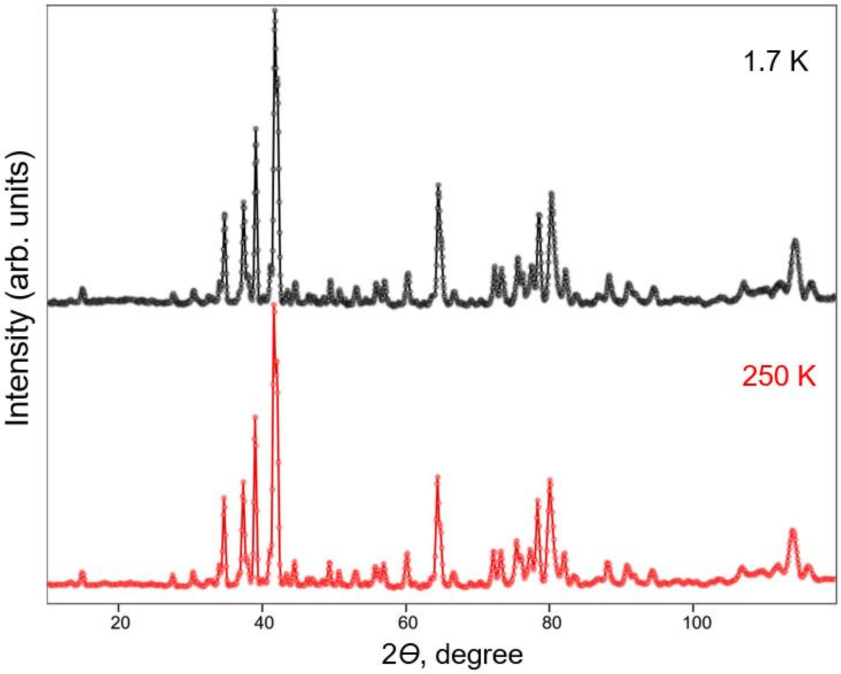

Figure S5. Powder neutron diffraction patterns of $Ba_4RuMn_2O_{10}$ at 1.7 and 250 K.

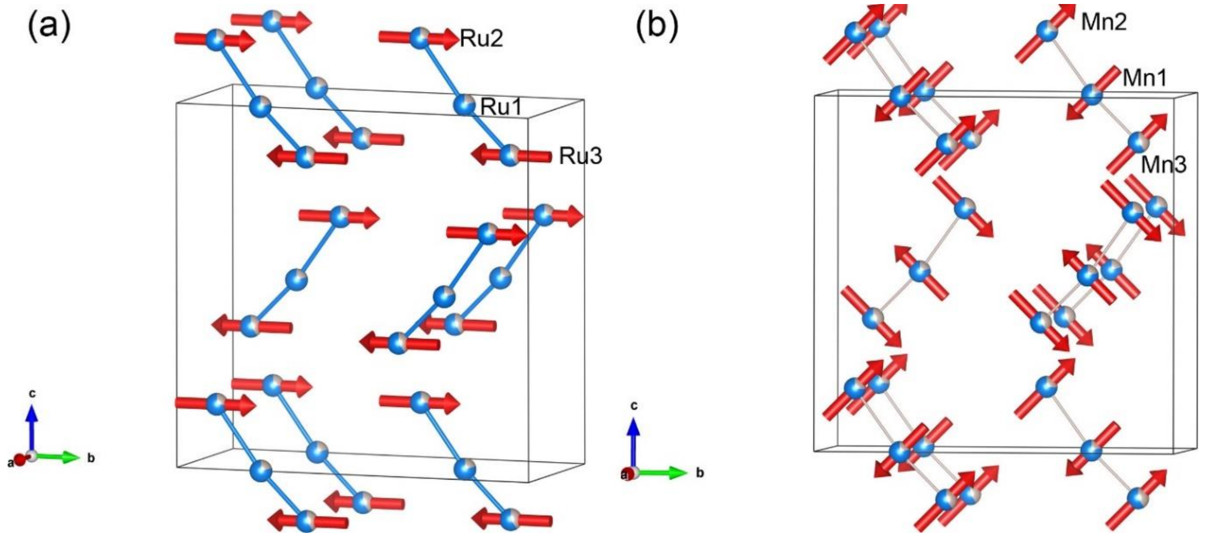

Figure. S6. Magnetic structures reported for the parent compounds $Ba_4Ru_3O_{10}$ (a) and $Ba_4Mn_3O_{10}$ (b) redefined with the polar symmetry of $Ba_4RuMn_2O_{10}$. The magnetically ordered compounds can be described using the magnetic space group *Cc'm*2$_1$. In $Ba_4Ru_3O_{10}$, the Ru2 and Ru3 sites have antiparallel moments, while the Ru1 site has no magnetic moment. For the Mn, all three sites are ordered with the moments of Mn2 and Mn3 parallel and Mn1 antiparallel.

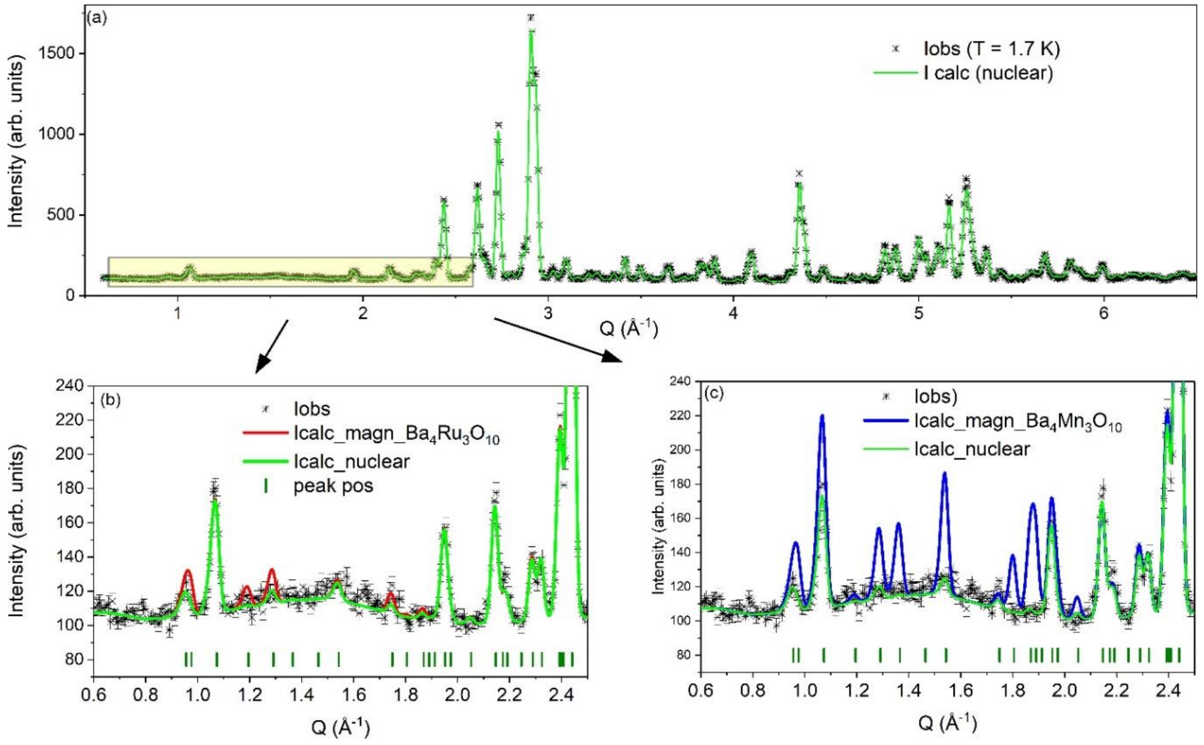

Figure S7. (a) The refined neutron data of $Ba_4RuMn_2O_{10}$ measured at 1.7 K, assuming only a nuclear contribution (green line in a) and low angle ranges with the simulated magnetic Bragg intensities for the two parent compounds $Ba_4Ru_3O_{10}$ (red line in b) and $Ba_4Mn_3O_{10}$ (blue line in c) with the adopted polar crystal structure of $Ba_4RuMn_2O_{10}$. Additional scattering is expected to appear at the (002), (130), and (131) reflections.
